# Biomechanical evaluation of a healed acetabulum with internal fixators: finite element analysis

**DOI:** 10.1186/s13018-023-03736-2

**Published:** 2023-03-27

**Authors:** Pengyun Duan, Xiaohong Ding, Min Xiong, Panfeng Wang, Shipeng Xu, Wei Du

**Affiliations:** 1 School of Mechanical Engineering, University of Shanghai for Science and Technology, Shanghai, People’s Republic of China; 2 Department of Orthopaedics, Changhai Hospital, Naval Medical University, Shanghai, People’s Republic of China

**Keywords:** Acetabulum, Internal fixation, Healed pelvis, Finite element analysis, Three-column theory

## Abstract

**Background:**

Treatment of complicated acetabular fracture with internal fixation usually has high risk of failure because of unbefitting fixation. However, evaluation of the biomechanical effect of internal fixation under physiological loading for fracture healing is still generally rarely performed. The purpose of this study is to analyze the biomechanical characteristics of a healed acetabulum with designed internal fixators under gait and to explore the biomechanical relationship between the healed bone and the internal fixator.

**Methods:**

A patient-specific finite element model of whole pelvis with designed internal fixators was constructed based on the tomographic digital images, in which the spring element was used to simulate the main ligaments of the pelvis. And the finite element analysis under both the combination loading of different phases and the individual loading of each phase during the gait cycle was carried out. The displacement, von Mises stress, and strain energy of both the healed bone and the fixation were calculated to evaluate the biomechanical characteristics of the healed pelvis.

**Results:**

Under the combination loading of gait, the maximum difference of displacement between the left hip bone with serious injury and the right hip bone with minor injury is 0.122 mm, and the maximum stress of the left and right hemi-pelvis is 115.5 MPa and 124.28 MPa, respectively. Moreover, the differences of average stress between the bone and internal fixators are in the range of 2.3–13.7 MPa. During the eight phases of gait, the stress distribution of the left and right hip bone is similar. Meanwhile, based on the acetabular three-column theory, the strain energy ratio of the central column is relatively large in stance phases, while the anterior column and posterior column of the acetabular three-column increase in swing phases.

**Conclusions:**

The acetabular internal fixators designed by according to the anatomical feature of the acetabulum are integrated into the normal physiological stress conduction of the pelvis. The design and placement of the acetabular internal fixation conforming to the biomechanical characteristics of the bone is beneficial to the anatomical reduction and effective fixation of the fracture, especially for complex acetabular fracture.

## Introduction

The acetabulum is one of the most important bone in the human body, which undertakes the complex physiological function of weight-bearing walking. Complicated acetabular fractures caused by high energy and severe violence are often accompanied by high disability and mortality. At present, open reduction and internal fixation are often used to treat fracture in clinic [1, 2], and according to specific fractures, different internal fixation methods, such as screws, plates, and intramedullary nail, are applied. Because the acetabulum is a unique structure with irregular shape and complicated surrounding anatomical relation, and deep fracture often occurs with various injuries, the treatment of complicated acetabular fractures usually has high risk of failure. The loss of reduction and complications caused by improper internal fixation often occur, which seriously affect the life quality of patients [3–5]. The main reason for improper fracture fixation is the unfitting structure of internal fixator and its fixation method. In terms of biomechanical view, the design and placement of internal fixator should conform to the physiological force of the bone, so that the broken bone can be anatomical reduction and stable fixation, and restore the stress conduction of the bone. Hence, to improve treatment effect of fracture, it is of great significance to study biomechanical characteristic of fracture acetabulum with internal fixation.

Existing biomechanical researches on fracture internal fixation are usually using either experimental testing or finite element analysis. Biomechanical studies of different fixation methods are important to determine optimal fixation techniques and test novel implants in a preclinical setting [6]. Cai et al. [7], Fan et al. [8] and Marmor et al. [9] adopt cadaveric bone for biomechanical testing, which leads to difficulty in obtaining experimental samples. Krappinger et al. [10, 11], Alfonso et al. [12] and Wu et al. [13] choose synthetic pelvis models, which leads to high cost. Additionally, there is no existing standardized mechanical experimental method [1, 6]. Finite element analysis is a powerful tool for orthopedic biomechanical research [14, 15]. Kocsis [16], Bignardi et al. [17], Hedelin et al. [18], Zhang et al. [19] and Lei et al. [20] construct three-dimensional model of bone and fracture internal fixation based on CT data and numerical simulation is carried out, which is low cost, high repeatability and accurate results, but involves the problem of how to validate the finite element model and the applied loading. The existing biomechanical study of fracture internal fixation can provide some guidance for fracture treatment, but there are also some common problems, which arise from the following aspects.

1. Most of the physiological loads simulated in the current studies are relatively simple, focusing on one-legged or two-legged stance.
2. The evaluation criteria of fixation methods, is usually the maximum fixation stiffness, which is limited.
3. The relationship between the biomechanical characteristics of fracture internal fixation and the effect of bone healing is still not clear.

To evaluate the implantation effect of fixation method and explore the relationship between the biomechanical characteristics of internal fixation and the effect of bone healing, it is of great value to analyze the internal fixation implants and their fixation methods with good clinical results [21]. Such analysis not only helps understand the principle of fixation, but also provide guidance for design of internal fixation implants and precise treatment of fractures. However, due to the complexity of internal fixation of acetabular fracture, there is little biomechanical analysis and further research on the clinical cured cases. Therefore, this is a novel work presenting the in-depth biomechanical analysis of cured complex acetabular fracture and study on clinical internal fixation implant and fixation method.

A pelvis of a cured patient with complex acetabular fracture is selected as the research object in this paper. Three-dimensional model of healed pelvis with internal fixators was established by using three-dimensional reconstruction technique, and the finite element analysis under both the combination loading of different phases and individual loading of each phase during the gait cycle was carried out. The aims of the study are: (1) analysis of the biomechanical characteristics of acetabular internal fixator in the treatment of acetabular fractures under gait cycle loading, obtaining some relevant mechanical indexes such as deformation, stress and strain energy, and (2) exploration of the relationship between the biomechanical characteristics of internal fixation and the healing effect of acetabular fractures, so as to provide guidance to accurate treatment of acetabular fractures.

## Materials and methods

This study was approved by the ethics committee of Changhai Hospital of Shanghai, China. Written informed consent was obtained from the patient prior to the study.

### Surgical techniques

**Fig. 1:**
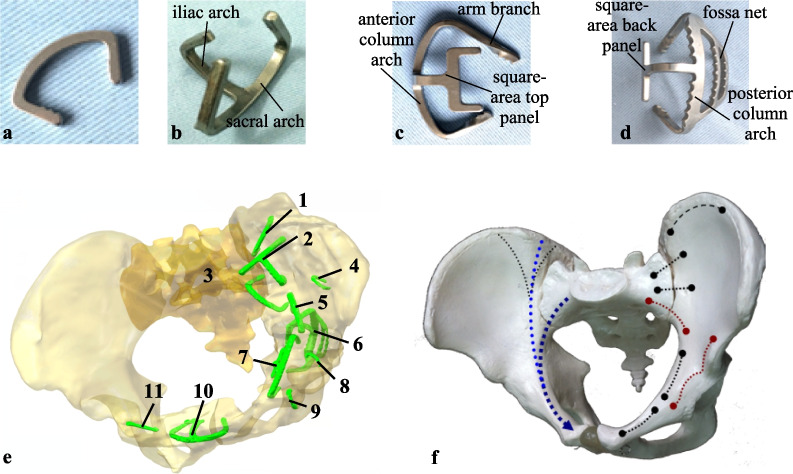
Acetabular internal fixators and acetabular force line and placement of internal fixators: **a** bow-teeth nails, **b** sacroiliac joint three-corner fixator, **c** anterior column or wall fixator, **d** posterior column or wall fixator, **e** placement of acetabular internal fixators, **f** force line and point of acetabular anterior column [23]

A 33-year-old female patient (height 162 cm, weight 55 kg) suffered a serious injury in the left acetabulum, multiple fractures and a slight injury in the right acetabulum due to a car accident. According to the fracture condition of the patient, the acetabular internal fixators were placed and no longer removed. The acetabular internal fixators used in this patient are designed by the variations of pelvic anatomy and anatomical feature of the acetabulum [22]. They are classified into three groups: bow-teeth nails for connection of two displaced fragments (Fig. 1a, b), anterior column or wall fixator (Fig. 1c), and posterior column or wall fixator (Fig. 1d). There are eleven internal fixators which is numbered 1–11 clockwise (Fig. 1e), including seven bow-teeth nails, one sacroiliac joint three-corner fixator connected by iliac arch and sacral arch, two anterior column or wall fixators composed of anterior column arch, arm branch and square-area top panel, and one posterior column or wall fixator composed of posterior column arch, fossa net and square-area back panel. In order to provide good anatomical reduction and stability, the acetabular internal fixators are mostly placed in the force line and point of acetabular anterior column by the doctor’s experience [23] (Fig. 1f), for example, fixators 2 and 3 are placed in the anterior of the sacroiliac joint, and the fixators 5 and 7 are placed at the arcuate line (Fig. 1e). The patient was cured after acetabular internal fixation, and the left acetabulum with severe injury achieved integrity reduction.

### Geometric modeling of pelvis and internal fixators

The healed pelvis and acetabular internal fixators models were constructed using computer tomography (CT) data (with 1 mm slice thickness, including 376 images). The CT scanning data were imported into Mimics 14.0 software (Materialise, Belgium) to reconstruct the surface geometry models of the healed pelvis and internal fixators by region growing and mask editing. Semi-automatic segmentation of the CT data was performed to identify the boundary of sacrum, left and right hip bone by contour interpolation. The surface geometry models were exported as point cloud format files. Then these files were imported into Geomagic Studio 12 (Geomagic, USA) for smoothing and surface construction and modification, and converted into solid models, respectively.

### Finite element model

The solid models of healed pelvis and internal fixators were meshed using HyperMesh 14.0 (Altair, USA). Due to the same CT images, the spatial position of the bone and the internal fixators is fused automatically. Additionally, the model was processed by Boolean operation, so the mesh of the contact between bone and internal fixators were set in the form of common nodes. The bone consists of cancellous and cortical parts in the finite element model. Solid elements are used for cancellous bone and internal fixators. Shell elements are clad around the solid elements to represent the cortical bone. The thickness of the cortical bone is assumed to be an average value of 1.5 mm [24]. The finite element model consists of 11,807,170 tetra4 solids elements and 1,374,437 tria3 shell elements (Table 1), for an average element size of 0.5 mm. The model is defined as linear elastic, homogeneous and isotropic [14, 15, 25]. The material properties are listed in Table 1 [24, 26].

**Table 1:** Material properties of hip bones and fixators [24, 26]

	Elasticity modulus (GPa)	Poison ratio	Thickness (mm)	Element number	Node number
Cortical bone	17	0.3	1.5	1,374,437	687,270
Cancellous bone	0.7	0.2		11,388,380	2,257,826
NiTi alloy	110	0.32		418,790	99,940

**Fig. 2:**
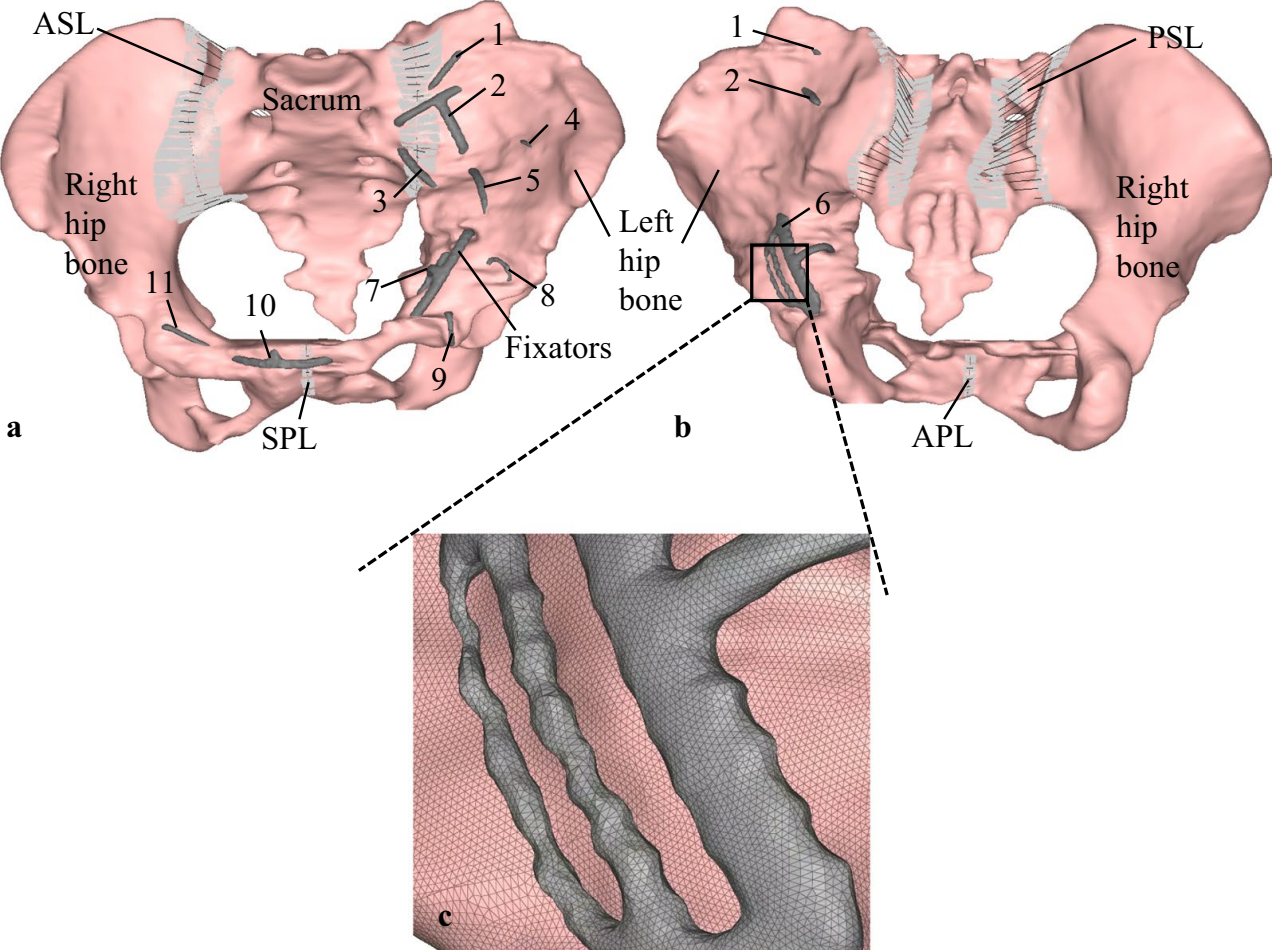
Finite element model of healed pelvis of the patient with acetabular internal fixators: **a** anterior view, **b** posterior view, **c** zoom view

The pelvis includes the left and right hips and sacrum to form a complete pelvic ring. The pelvis is a stable ring structure due to the strong support of the sacroiliac ligament and the firm ligament connection at the pubic symphysis. The main ligaments, including the anterior and posterior sacroiliac, the superior and arcuate pubic, are modeled as linear spring elements with tension-only stiffness in finite element models, as shown in Fig. 2. The rigid elements are applied to ligament attachment area nodes based on the anatomical feature of the pelvis. The stiffness values for ligaments are estimated from existing literature [27, 28] (Table 2). The tied contact pairs at the sacroiliac joint and pubic symphysis are set in finite element model.

**Table 2:** Material properties of the pelvic ligaments in the model [27, 28]

Ligaments in the model	Stiffness (N/mm)	Number of springs
Anterior sacroiliac ligament (ASL)	5000	20 × 2
Posterior sacroiliac ligament (PSL)	5000	20 × 2
Superior pubic ligament (SPL)	500	5
Arcuate pubic ligament (APL)	500	5

### Loading and boundary conditions

**Fig. 3:**
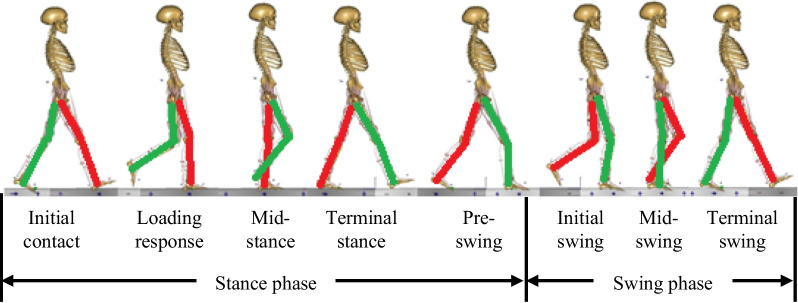
Typical gait phases of the human (the left side is in red, the right side is in green)

**Fig. 4:**
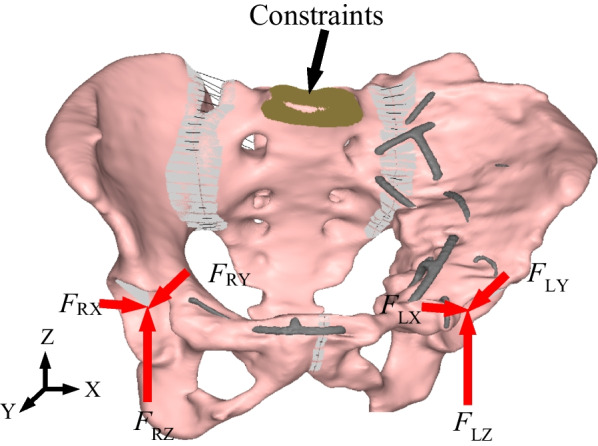
Constraints and the hip contact force vector

Gait is one of the most common and essential activities in daily human life, periodic and regular. Each gait cycle consists of a sequence of ordered gait phases. Figure 3 shows the typical gait cycle includes eight phases of a single leg, starting from heel strike of the left leg and continuing until that heel strikes again [29]. Eight phases are initial contact (IC), load response (LR), mid-stance (MST), terminal stance (TST), pre-swing (PSW), initial swing (ISW), mid-swing (MSW), and terminal swing (TSW). The description and proportion of each phase of the gait cycle is listed in Table 3. The hip joint forces experienced by the pelvis for eight phases of gait are obtained using an existing healthy model (62 kg, 173 cm) which is freely available from the AnyBody software (AnyBody Technology, Denmark). It should be noted that the existing healthy model in AnyBody is different from the specific-patient in finite element modeling, however, because the focus of our study is on the distribution rules and the relative values of biomechanical response between the concerning parts, rather than the magnitude of them, it is acceptable for using the hip joint forces directly from the analysis results from AnyBody. The magnitude of the hip joint forces for the eight phases of the gait cycle is listed in Table 4. The direction of the hip joint forces is shown in Fig. 4. From the comparison, it is found that the trend of the hip joint force in the eight phases of the gait cycle obtained here is similar to that of literature [29]. Figure 4 shows the force vectors are applied on the node at location consistent with the femoral head. The node is attached to the acetabulum by the rigid element, which means the forces are applied to the relevant areas of the acetabulum. For the boundary condition, the nodes located on the superior surface of the sacrum were constrained in all DOF (Fig. 4).

**Table 3:** The description of each phase of the gait cycle [29]

Phase/subcase	Description	Percent walking cycle (%)	
IC	Double support, weight shift beginning to left foot		2
LR	Beginning left single support, right swing phase		13
MST	Midstance, left single support phase		35
TST	Terminal stance, beginning right heel strike		48
PSW	Double support, weight shift beginning to right foot		52
ISW	Beginning right single support, left swing phase		63
MSW	Midswing, right single support phase		85
TSW	Terminal swing, beginning left heel strike		98

**Table 4:** Applied forces of hip joints

Phase	Left side				Right side			
	*F*_LX_/N	*F*_LY_/N	*F*_LZ_/N	*F*_L_/N	*F*_RX_/N	*F*_RY_/N	*F*_RZ_/N	*F*_R_/N
IC	− 599.8	338.7	1665.4	1802.2	411.4	− 125.08	549.39	697.7
LR	− 538.2	148.6	1761.4	1847.7	132.91	− 24.82	204.13	244.9
MST	− 296.0	− 74.8	1215.2	1253.0	52.62	7.84	45.2	69.8
TST	− 454.3	− 485.6	2064.8	2169.2	185.1	213.59	501.73	575.9
PSW	− 350.7	− 450.2	1526.1	1629.3	175.06	354.94	761.55	858.3
ISW	− 217.1	− 141.7	399.4	476.1	617.72	428.18	1776.51	1929.0
MSW	− 43.4	− 6.1	31.4	53.9	278.32	− 18.42	1021.09	1058.5
TSW	− 93.6	187.2	416.8	466.4	596.08	− 429.94	1901.74	2038.8

*F*_L_ represents left hip joint force, *F*_R_ represents right hip joint force, X, Y and Z represent axis

### Mesh sensitivity analysis

**Fig. 5:**
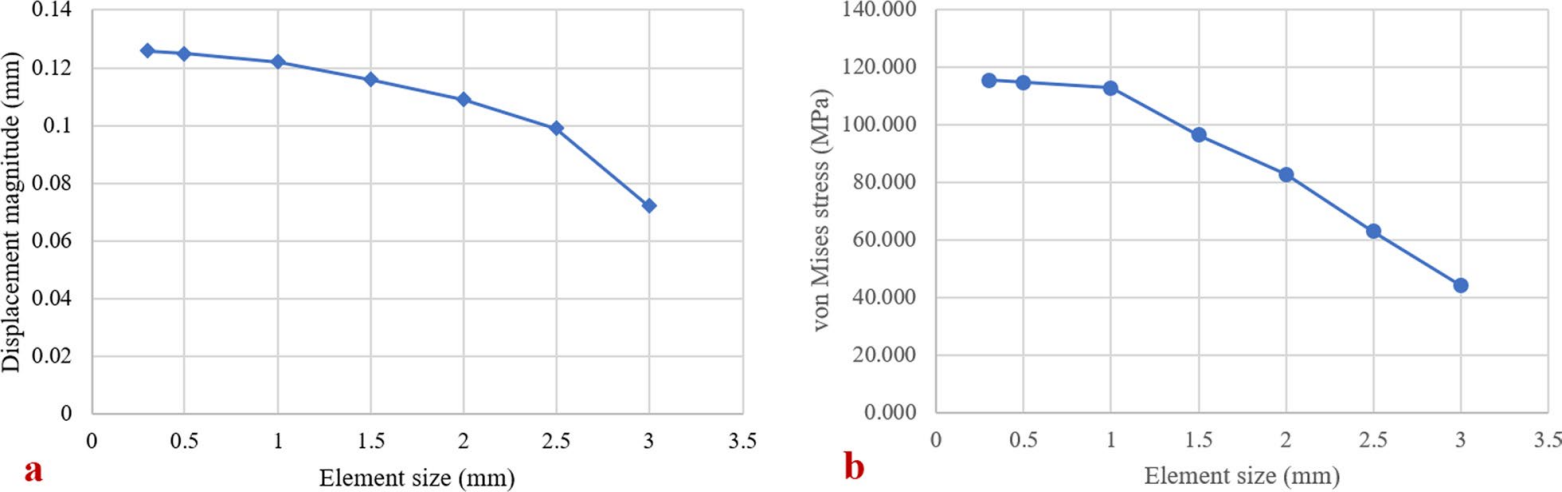
Mesh sensitivity analysis: **a** convergence curve between the displacement and the element size, **b** convergence curve between the von Mises stress and the element size

A mesh sensitivity analysis was carried out using the model of right acetabulum with different element size. The result of mesh sensitivity analysis is shown in Fig. 5. When the element size decreases from 3 to 1 mm, the value of displacement and stress increases greatly. However, when the element size decreases from 1 to 0.3 mm, the increase of displacement and stress is small. The sensitivity analysis shows that the convergence is achieved. Considering the geometric complexity and computational efficiency of the bone model, the element size 0.5 mm is selected for the finite element models in our study.

## Results

To investigate the effect of internal fixators on acetabular fracture reduction and pelvic stability, the deformation, von Mises stress, and strain energy of the whole pelvis and the healed acetabulum with internal fixators are analyzed under eight gait cycle phases or a combination of all phases of the gait cycle. Note that the combination of all phases is composed of eight different phases in proportion (the corresponding weight factor is 0.11, 0.22, 0.13, 0.04, 0.11, 0.22, 0.13, 0.04, respectively) according to the occurrence of each phase in gait cycle (Table 3 lists the percentage [29]). The main purpose of this study is to analyze the biomechanical characteristics of the healed left hip bone after internal fixation; therefore, the right acetabulum with minor injury is compared as a relatively healthy bone approximately.

### Displacement analysis

**Fig. 6:**
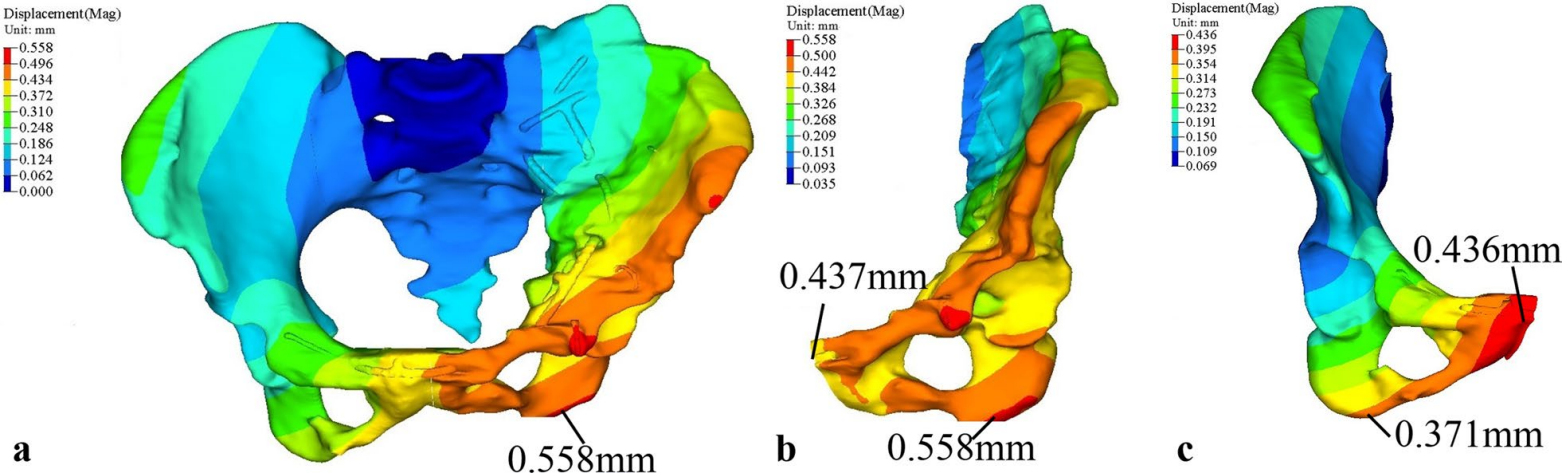
The displacement of pelvis with internal fixators under the combination of all phases of the gait cycle: **a** the pelvis with internal fixators, **b** the left healed hemi-pelvis, **c** the right healthy hemi-pelvis

Figure 6 shows the displacement of pelvis with internal fixators under the combination of all phases of the gait cycle. The maximum displacement in the pelvis is 0.558 mm appears at the left hemi-pelvis near the ischial tuberosity (Fig. 6a). The displacement distribution in the left and right hemi-pelvis shows a slight variance. The maximum displacement of the right hemi-pelvis is 0.436 mm on the pubic symphysis (Fig. 6c). In comparison, the left acetabulum moves slightly upward after healing. In some gait cycle phases, the left hip joint forces are greater than that of the right. The left hemi-pelvis produces more movement along pubis from ischial tuberosity.

### Stress analysis

**Fig. 7:**
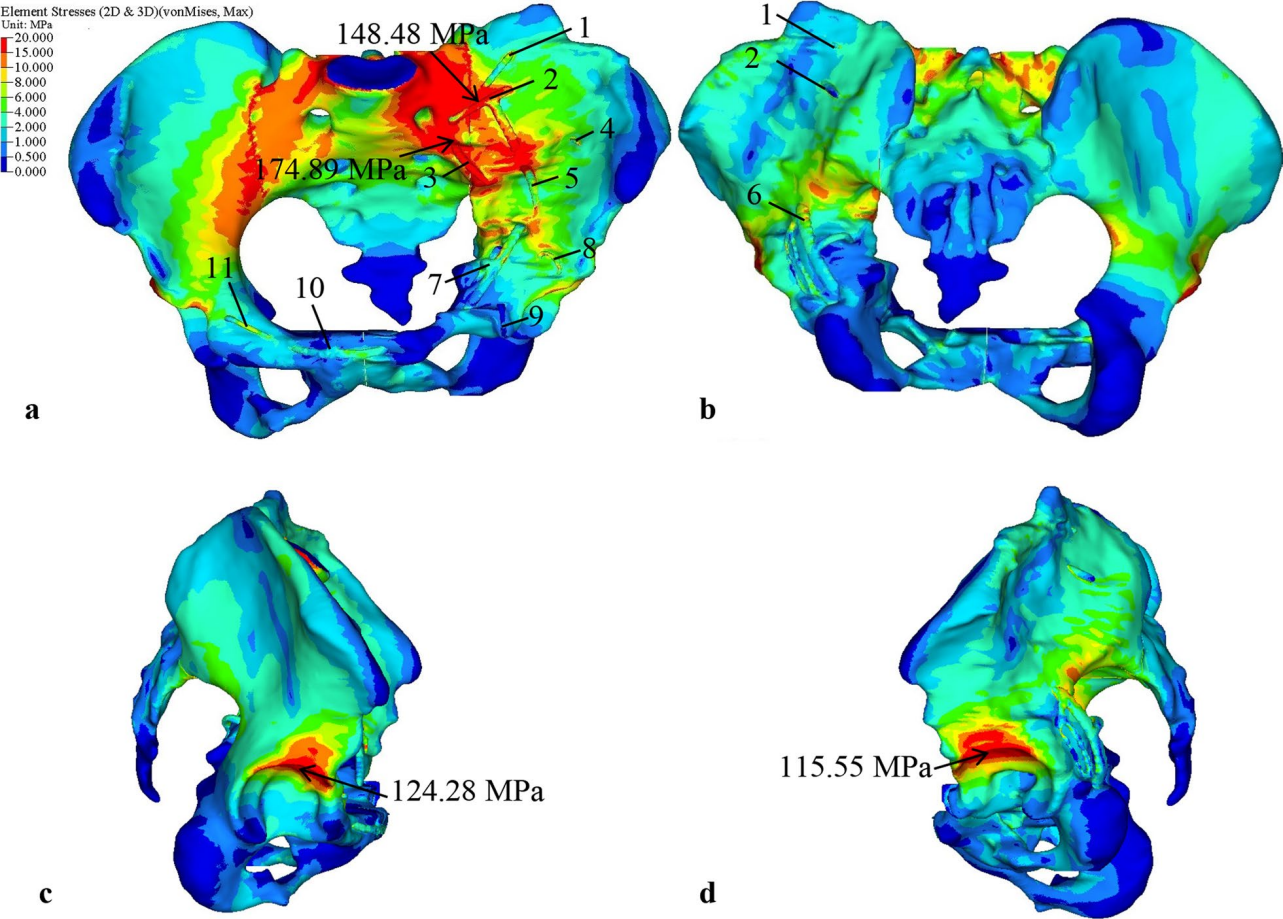
The von Mises stress distribution of pelvis with internal fixators under the combination of all phases of the gait cycle: **a** anterior view, **b** posterior view, **c** lateral view (from right to left), **d** lateral view (from left to right)

Figure 7 shows the von Mises stress distribution of the pelvis with internal fixators under the combination of all phases during the gait cycle. The high-stress regions are concentrated in the superior of the acetabulum, sacroiliac joint, the iliac arch of the arcuate line, and the great notch of the ischium (Fig. 7). A maximum stress of 174.89 MPa is observed at fixator 3 intersection with the sacral cortex. The secondary peak stress of 148.48 MPa is shown on the fixator 2 intersection with the iliac cortex. The results indicate that the peak stress is less than the yield strength (1050 MPa) and fatigue strength (315 MPa) of titanium alloy [19], and no failure occurs. The pelvic bone shows the maximum stress of 124.28 MPa at the acetabular fossa of the right hemi-pelvis, while the maximum stress of the left hemi-pelvis is 115.55 MPa on the acetabular fossa, which decreases by roughly 7% compared with that of the right hemi-pelvis.

**Fig. 8:**
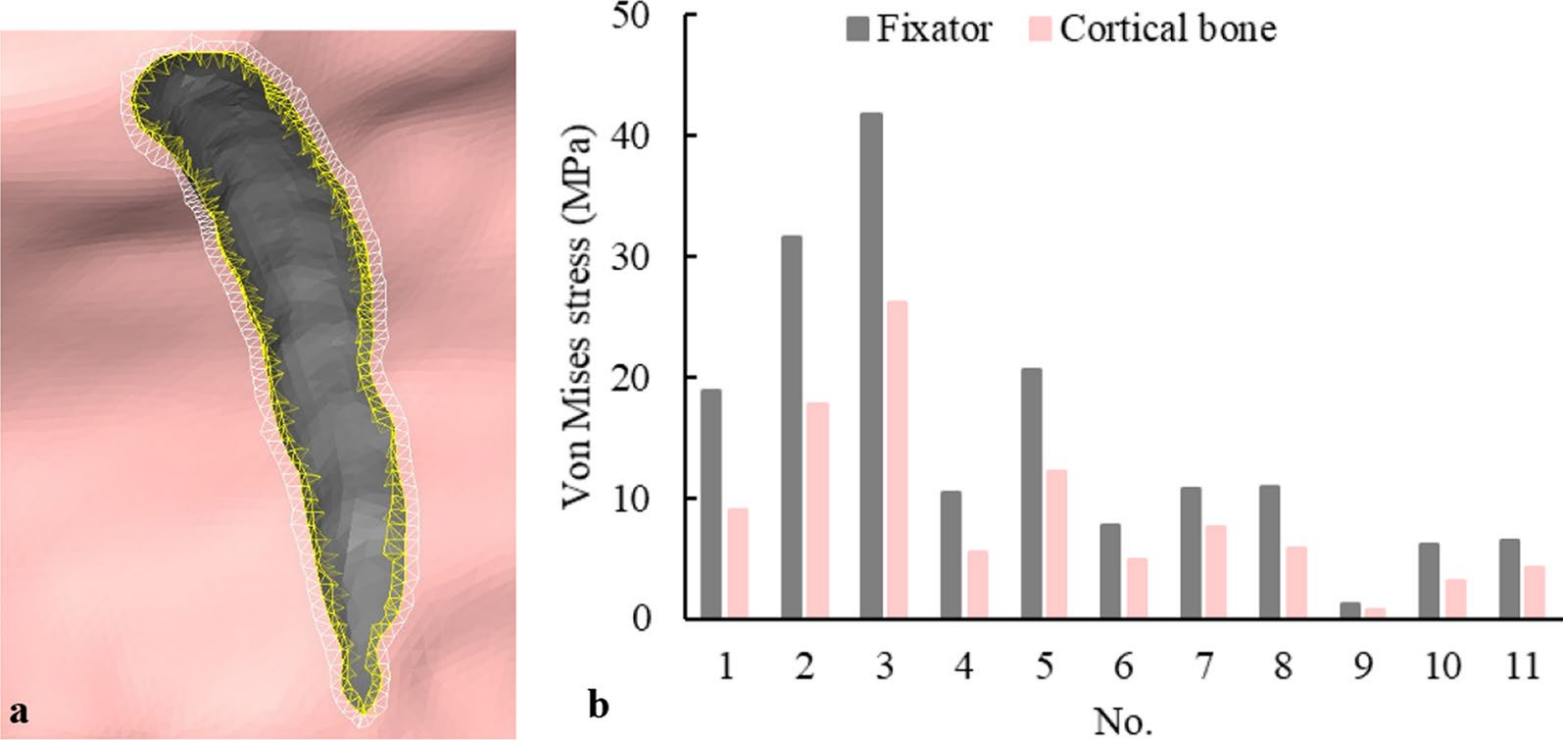
Elements selection example and comparison of von Mises stresses at selected elements under the combination of all phases of the gait cycle: **a** element selection example, **b** comparison of von Mises stresses at selected elements under the combination of all phases of the gait cycle

To compare the stress on the bone and that on the fixator, the elements at the connection of internal fixators and bone are selected to calculate the average stress. Figure 8a shows the selected elements in fixator 5, where the yellow brightening elements are the selected elements in fixator 5, and the white brightening elements are the iliac cortical elements in contact with fixator 5. Figure 8b shows comparison of von Mises stresses on the selected elements under the combination of all phases during the gait cycle. It is found that stress on the internal fixators is higher than that on the bone, because the elastic modulus of the fixator is higher than that of bone. However, the stress differences between the bone and the fixators are not too large; for example, the average stress differences between the fixators and the bone are 13.7 MPa, 9.9 MPa, and 8.4 MPa in the fixator 2, 1, and 5, respectively, and the average stress difference between other fixators and bone are roughly 2.3–5.2 MPa. The results indicate that the position of the fixator impacts the stress distribution, but the stress shielding of acetabular internal fixation is minor. The biomechanical compatibility between fixator and bone is good.

**Fig. 9:**
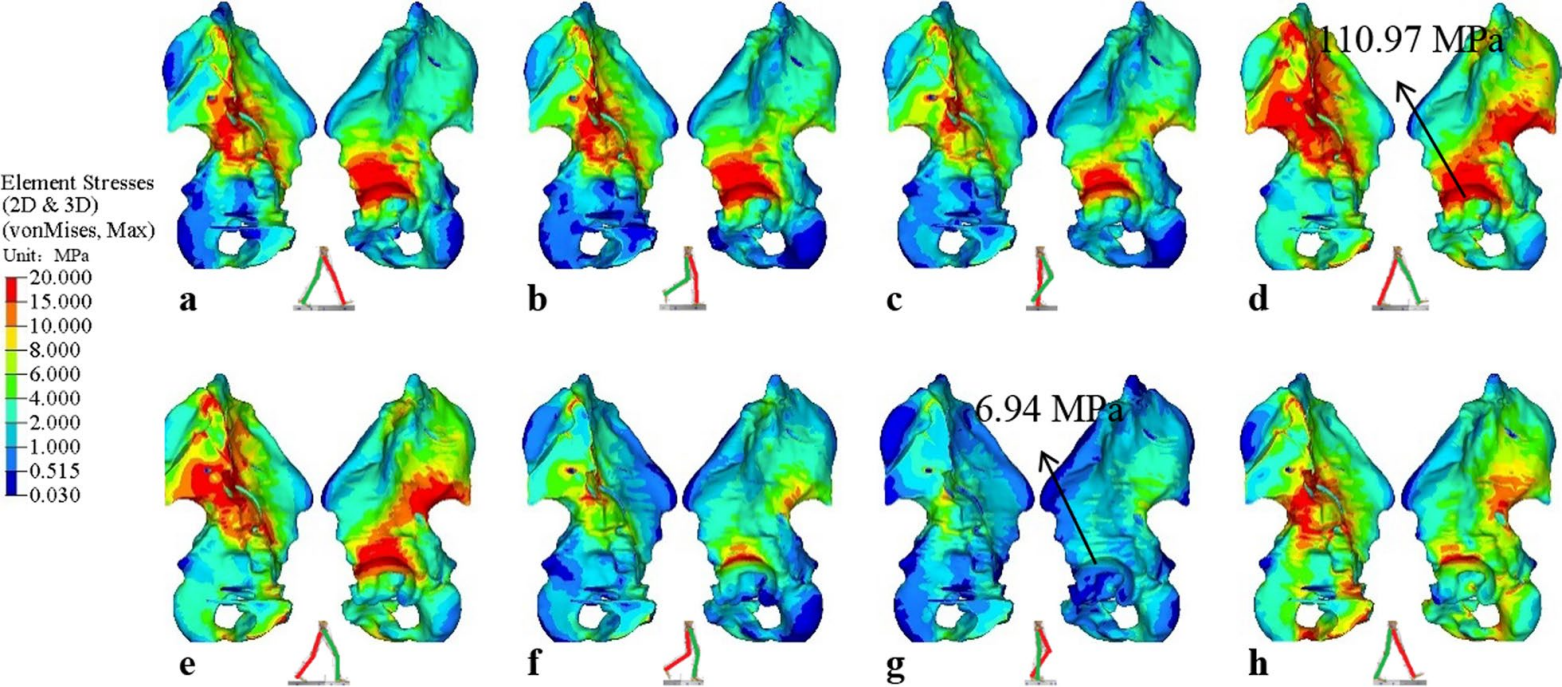
Von Mises stress distribution of the left hemi-pelvis under each phase of gait cycle: **a** initial contact phase, **b** loading response phase, **c** mid-stance phase, **d** terminal stance phase, **e** pre-swing phase, **f** initial swing phase, **g** mid-swing phase, **h** terminal swing phase

Figure 9a–h shows the von Mises stress distribution of the left hemi-pelvis for each phase of the gait cycle, respectively, with the corresponding gait cycle indicated at the bottom center of each figure. During the stance phase, the left leg changes from flexion to extension. When flexion, the stress distribution on the lunate surface, superior acetabular dome, and iliac arch of the arcuate line are high (Fig. 9a, b). When extension, in addition to the previous observation, the stress distribution also displays on the sacroiliac joint and greater sciatic notch (Fig. 9c–e). During the swing phases, the left leg is all flexion, a noticeable stress reduction is observed around the lunate surface, superior acetabular dome, and the sacroiliac joint (Fig. 9f–h). On the other hand, the high-stress regions decrease during the swing phases. In the TST phase, with the left foot single support, the larger the extension angle, the greater is the joint force (the peak force magnitude of approximately 2169N), thus the maximum stress is 110.97 MPa. In the MSW phase, with the left foot in the air (the force magnitude of approximately 54N), the maximum stress gradually decreases to 6.94 MPa. From the medial view of the pelvis, the stress distribution is mainly transmitted from the acetabulum along the arcuate line upward to sacroiliac joint. From the lateral view of the pelvis, the stress distribution is transmitted from the acetabulum upward to anterior inferior iliac spine during flexion, and along the greater sciatic notch to posterior inferior iliac spine during extension. The results indicate that the stress distribution and conduction of the pelvis under gait are related to the biomechanics of the hip joint during walking. The hip joint plays an important role in the weight-bearing, multi-directional movement and balance function of the lower limbs of the human body.

**Fig. 10:**
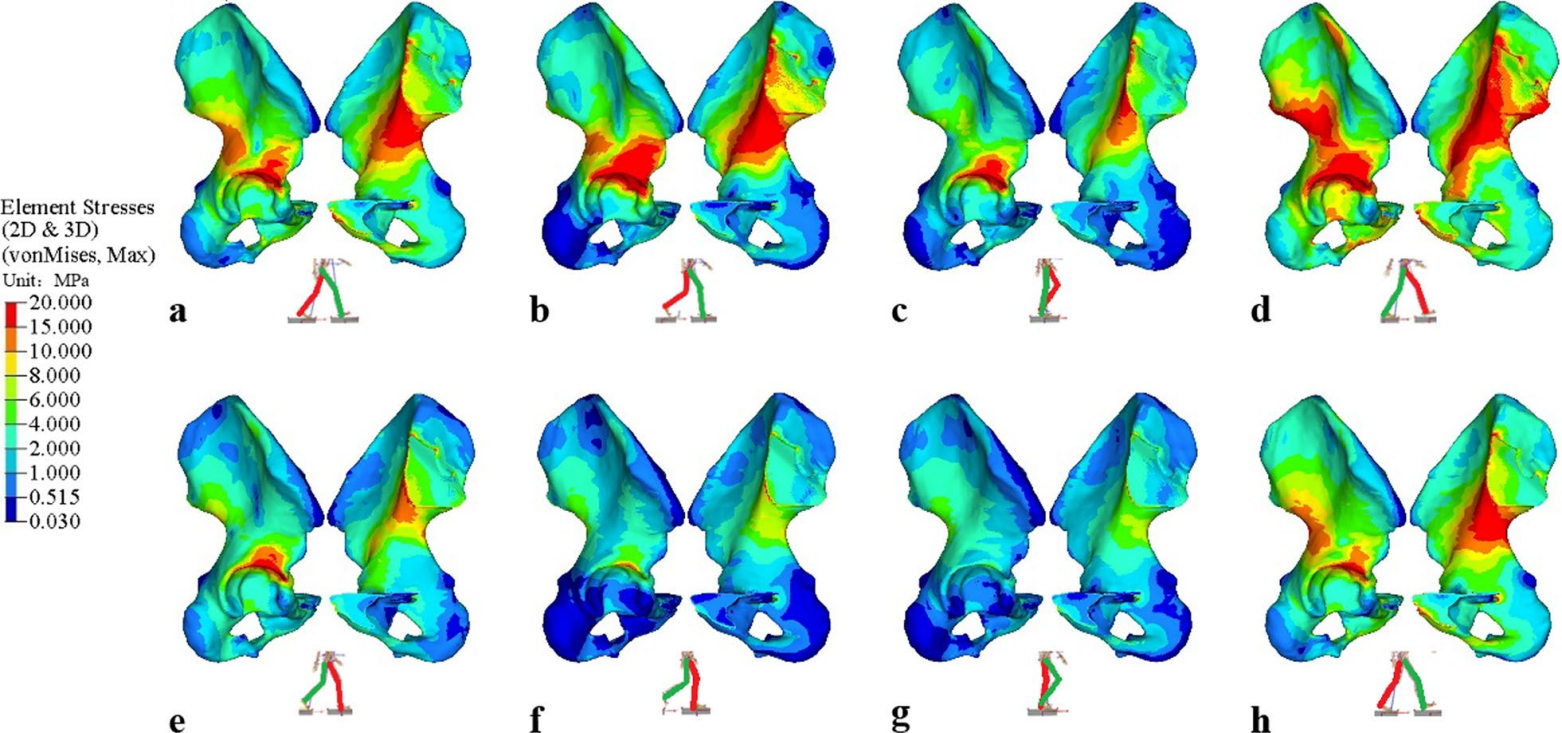
Von Mises stress distribution of the right hemi-pelvis under each phase of gait cycle: **a** initial contact phase, **b** loading response phase, **c** mid-stance phase, **d** terminal stance phase, **e** pre-swing phase, **f** initial swing phase, **g** mid-swing phase, **h** terminal swing phase

Human walking is a coordinated movement of alternating left and right legs. As shown in Fig. 3 and Table 3, when the left leg is at IC phase, the right leg is at PSW phase, and similarly for the other phases. In comparison, Fig. 10a–h shows the von Mises stress distribution of the right hemi-pelvis for each phase of the gait cycle, with the corresponding gait cycle indicated at the bottom center of each figure. Under the PSW phase, it is observed that the left hemi-pelvis and right hemi-pelvis produce a greater variation of stress distribution (Figs. 9e, 10e), and the right hemi-pelvis displays noticeably stress reduction. Similar observation can be found in IC phase (Figs. 9a, 10a). For the rest of the gait phases, small differences can be observed between left and right hemi-pelvis. Because the left and right legs are not completely symmetric in simulation, there are significant differences in obtained right hip joint forces under the IC and PSW phases. The results indicate that the stress distribution of the left hip bone returns to normal during gait cycle.

### Strain energy analysis

**Fig. 11:**
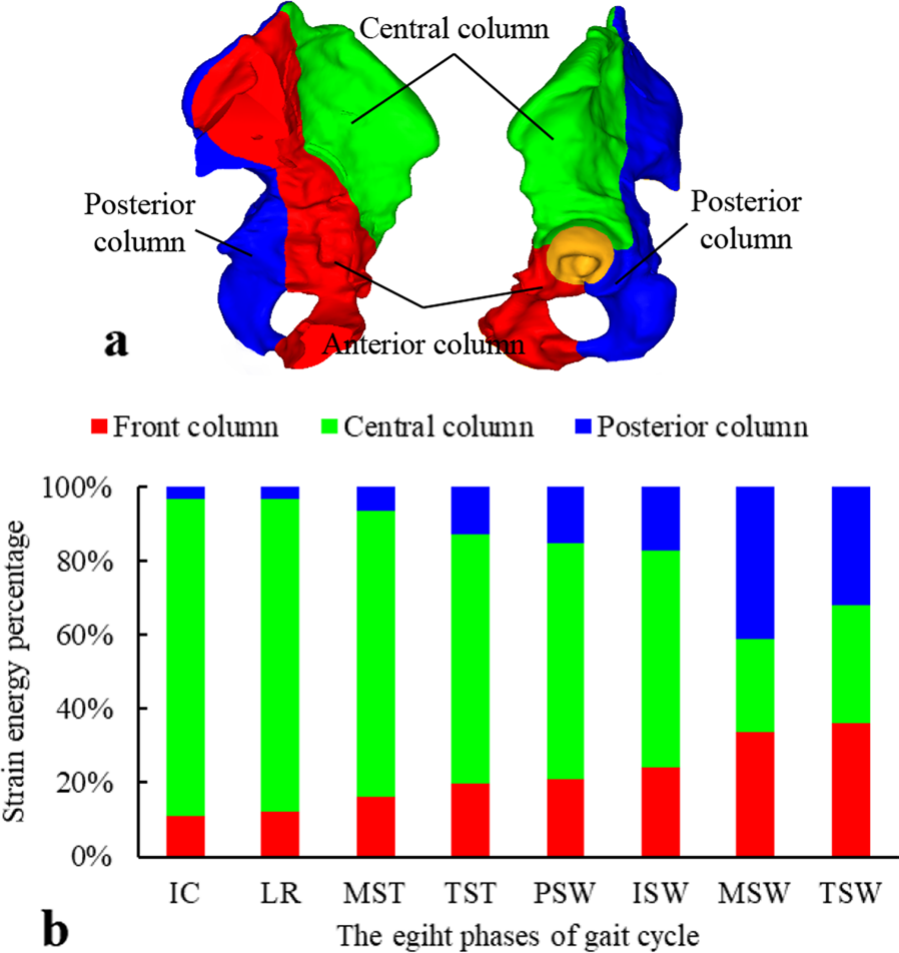
The acetabular three-column and strain energy ratios: **a** the components of the acetabulum in 3-column classification are marked with different colors, **b** strain energy ratio of acetabular three columns in the eight phases of the gait cycle

In order to guide the reduction and fixation of acetabular fractures, Zhang et al. [30] put forward the acetabular three-column theory from the view of acetabular anatomy and the practical treatment of acetabular fractures and combined with a large number of clinical experiences. According to the acetabular three-column theory, the anterior column is formed with the cortical thickening region of the ilium and the pubic bone migration, the central column is formed with the migration of the acetabular roof in loading-bearing direction, and the posterior column is formed with the sciatic migration. The left acetabulum is divided into the above three regions, as shown in Fig. 11a, in which the red region is the anterior column, the green region is the central column, and the blue region is the posterior column. When a structure is deformed under the action of external force, the strain energy is stored inside the structure. With strain energy as the mechanical index, the load-bearing capacity of structure can be quantitatively analyzed from the view of energy flow. Combined with the acetabular three-column theory, in order to explore the load-bearing capacity of the three-column during the gait, the percentages of the strain energy of the three columns in the total strain energy under eight phases of gait cycle are calculated according to Eq. (1),

1 $$\begin{aligned} E & = E_{{\text{a}}} + E_{{\text{c}}} + E_{{\text{p}}} \\ \mu_{i} & = \frac{{E_{i} }}{E}(i = {\text{a, c, p}}) \\ \end{aligned}$$

where *E*_a_, *E*_c,_ and *E*_p_ are the strain energy of the anterior, central, and posterior columns, respectively. *E* is the total strain energy of the three columns, and *μ*_a_, *μ*_c,_ and *μ*_p_ are the strain energy ratios of the anterior, central and posterior columns, respectively.

Strain energy ratios of three columns in the left acetabulum during the eight phases of the gait cycle are calculated by Eq. (1) and shown in Fig. 11b. It is found that the strain energy ratio of the central column decreases, and the anterior and posterior column increase gradually from the gait phase of IC to MSW, and the strain energy ratios of the central column are higher than that of the anterior and posterior column. The strain energy ratio of the central column in IC phase is closer to LR phase with the leg all flexion. From MST to PSW phase, the larger is the extension angle, the less is the strain energy ratio of the central column. In the swing phase, where the left leg changes from extension to flexion, the strain energy ratio of the central column decreases at first and then increases. In the MSW phase with the left foot in the air, the strain energy ratio of the central column is the smallest.

## Discussion

Open reduction and internal fixation are the most common method for the treatment of acetabular fractures. However, for complex comminuted acetabular fractures, it is difficult to select single reconstruction plates and screws to achieve effective fixation and functional recovery of acetabular fractures. Zhang et al. creatively developed the acetabular internal fixators according to the anatomical characteristics of the acetabulum and studied the application by retrospective analysis and follow-up of clinical results [22]. Retrospective analysis or follow-up method based on medical imaging data combined with scoring system and criteria to evaluate the recovery of patients is often used by clinicians [31–33], which is relatively reliable, but the workload is large. It requires a large number of case statistics to guide fracture treatment by using this method, which is poor in timeliness. Based on the finite element modeling and analysis of a patient-specific pelvis with acetabular internal fixators, this study analyzed the biomechanical characteristics of acetabular internal fixators in the treatment of acetabular fractures and explored the biomechanical relationship between the healed bone and the internal fixator.

The healed pelvis model with internal fixators was established by the finite element method, and cortical bone and cancellous were distinguished. Spring elements were used to simulate the main ligaments of the pelvis. Finite element analysis was carried out for the evaluation of biomechanical response of the acetabular internal fixators under loads of the gait cycle. Watson et al. constructed a series of finite element models of the same human pelvis with different boundary conditions and compared the deformation, stress and strain of the hemi-pelvis under the 15% loading regime (15% of the gait cycle) and 48% loading regime (48% of the gait cycle). For the complete pelvic ring model, the region with the greatest deformation under both conditions was the ischial-pubic bone. The high stresses concentrated in the inferior ilium, along the superior regions of the acetabular rim, and around the sacroiliac joint [24]. Figure 9b, d shows the similar results, which indicates the finite element model in this study is relatively reliable.

Based on the displacement analysis of the pelvis with internal fixators under the combination of all phases during the gait cycle, it was found that there was a difference in the displacement distribution between the left healed hip bone and the right healthy hip bone because the left hip bone after the anatomical reduction is not the same as the right hip bone. However, clinical studies have found that the smaller the postoperative displacement of the pelvic ring, the better the stability of the internal fixation. If the displacement of the pelvic ring is less than the reduction of 1 cm, its long-term function will be improved [19]. This study shows that the maximum displacement of the whole pelvic ring is smaller than 1 mm, and the maximum displacement difference between the left and right acetabulum is 0.122 mm, which indicate that the stability of internal fixation is ideal, and the integrity of the acetabulum is restored.

When acetabular fractures are stabilized to achieve integrity reconstruction and anatomical reduction, the closer is the postoperative bone stress to the natural stress of healthy bone, the better is the clinical effect of the internal fixation [19]. In this study, the stress distribution of the left and right hemi-pelvis under the combination of all phases of the gait cycle is very similar, and the maximum stress difference between the left and right hemi-pelvis is only 7%. During different phases of the gait cycle, the stress distribution of the left and right acetabulum is also similar, only a slight difference in the high-stress region under IC and PSW phases. Furthermore, from the comparison of the average stress between each internal fixator and the left hip bone under the combination of all phases of the gait cycle, it is found that most differences are between 2.3–5.2 MPa, which indicates that the acetabular internal fixators provide a good mechanical environment and conforms to the stress conduction of the bone.

**Fig. 12:**
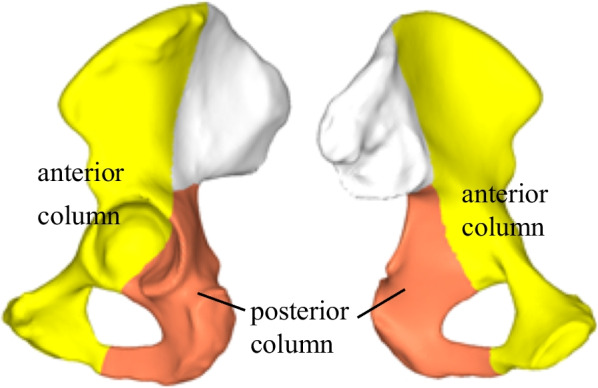
Diagram of components of the acetabulum on the two-column classification

Clinicians usually classify and treat acetabular fractures based on the two-column concept proposed by Judet-Letournel [34]. In the two-column concept of acetabulum, the iliac crest, spina iliace, pubic bone and the anterior acetabular rim are defined as the anterior column, while the ischium, ischial spine, the posterior acetabular rim and the hard ischial notch are defined as the posterior column (Fig. 12). However, with the in-depth study of acetabular fractures, there are limitations for complex acetabular fractures in two-column classification. Zhang et al. have put forward the three-column theory based on the physiological development and anatomical characteristics of the acetabulum and clinical experience, dividing the acetabular roof's weight-bearing area and the bone thickening area above it into the central column. The classification of acetabular fractures based on three-column theory more clearly reflects the severity of fractures and helps to provide guidance for anatomical reduction and fixation of acetabular fractures [30]. This study analyzes the load-bearing capacity of the three columns during gait by strain energy. The results indicate that the strain energy ratio of central column is higher than that of the anterior and posterior columns, which provides support for the acetabular three-column theory from the point of view of numerical quantification. Combined with the stress analysis results, the superior region of acetabulum generates higher stress (Fig. 7). Under different gait phases, the stress conduction of the pelvis shows a certain rule: on the medial side, the stress is mainly transferred upward from acetabulum through the arch line to the sacroiliac joint; on the lateral side the stress is mainly transmitted from the acetabulum forward to the anterior inferior iliac spine or backward through the great notch of the ischium to the posterior inferior iliac spine. These results are consistent with the firmness of the anatomical cortex of the acetabulum and the direction of the pattern. Therefore, the acetabular three-column classification is helpful for the reduction and fixation of acetabular fractures.

The quality of internal fixation and fracture healing are affected by postoperative activity. Any fixation methods of acetabular fractures need to be evaluated with the full pelvic ring under walking motions; thus, it is necessary to study the effects of walking with internal fixators attached so that the internal fixator can be better designed for earlier mobility [29]. However, the existing research is mainly focused on the finite element modeling and stress analysis of healthy pelvis under gait, and the evaluation of internal fixation methods rarely involves gait. Through the analysis of acetabular internal fixation during the gait cycle, it is found that the stress of the central and posterior column of the acetabulum is higher when the hip joint flexion, and the stress of the central and anterior column of the acetabulum is higher when the hip joint extension. Whenever flexion or extension, the maximum stress in the pelvis and internal fixators is less than the yield strength of the material. Therefore, it is suggested that extension training can be carried out earlier after using sacroiliac joint three-corner fixator for acetabular anterior column fractures. Flexion training can be carried out earlier after using the posterior column or wall fixator for acetabular posterior column fractures.

Although our results evaluated the biomechanical characteristics of the healed pelvis with internal fixators, simplifications and limitations of this study exist. First, unfortunately the material properties of the cortical and cancellous bone could not be accurately ascertained from the scan data, therefore homogeneous values from previous studies were used [24, 26]. In the existing studies, linear material is common and acceptable [29]. Second, the actual physiological environment of bone was simplified without considering the effect of cartilage in the model. However, as this study aims to evaluate the biomechanical characteristics of the healed pelvis with internal fixators, the cartilage is not the region of interest. Finally, because it is difficult to simulate the patient’s gait, the approximate joint forces of the left and right hip bones are adopted through the simulation of the healthy human in AnyBody. Additionally, the effect of pelvis muscle forces is not taken into consideration. The muscle appears to reduce stress concentrations, and produce a more even stress distribution on the pelvis [35]. The influence of the muscle forces on the pelvic stress conduction is not so great that it was not included in this study.

## Conclusions

In conclusion, this study successfully uses finite element analysis to evaluate the biomechanical characteristics of acetabular internal fixation for the treatment of a complex acetabular fracture. The results show the stress distribution of healed bone is different under each phase of gait cycle, the biomechanical evaluation of healed acetabulum with internal fixators under gait cycle is helpful for acetabular fracture fixation and functional training. It is also found that, under both the combination loading of different phases and the individual loading of each phase during the gait cycle, the stress distribution of the left and right hip bone is very similar, and the average stress difference between the bone and internal fixator is within a certain range. The acetabular internal fixation is integrated into the normal physiological stress conduction of the pelvis. The design and placement of the acetabular internal fixation conforming to the biomechanical characteristics of the bone is beneficial to the anatomical reduction and effective fixation of the fracture, especially for complex acetabular fracture.

It should be noted that although the patient-specific CT data were used to create the finite element model in this study, this is a special case. Although some mechanical rules can be revealed and some similar cases should be able to get more general conclusions, the proposed method is feasible and will be further explored in the future.

## Data Availability

The datasets used and/or analyzed during the current study are available from the corresponding author on reasonable request.
